# The Effect of Temporary Group Identity on Adolescent Social Mindfulness Decisions: An Empirical Study Using Team Sports Contexts

**DOI:** 10.3390/bs14110999

**Published:** 2024-10-27

**Authors:** Tao Tao, Wei Guo, Biye Wang

**Affiliations:** College of Physical Education, Yangzhou University, Yangzhou 225000, China; mx120240506@stu.yzu.edu.cn (T.T.); guowei@yzu.edu.cn (W.G.)

**Keywords:** prosocial behavior, group identity, intergroup bias, social mindfulness, team sport

## Abstract

Social mindfulness entails the consideration of the interests of others while respecting their autonomy. Although long-term group identity generates in-group favoritism in social mindfulness decisions, the effect of temporary group identity on social mindfulness remains to be validated. This study examined this effect by recruiting a convenience sample of 74 adolescents from a youth basketball club, who were randomly assigned to either an in-group or out-group decision-making condition. A basketball game scenario was used to establish temporary group identities, referencing the Minimal Group paradigm for grouping while applying the SoMi paradigm in a real-world context. The results showed that adolescents exhibited higher social mindfulness (χ^2^ = 22.774, *df* = 1, *p* < 0.001) and preference-adjusted social mindfulness (χ^2^ = 6.861, *df* = 1, *p* < 0.01) toward in-group compared to out-group members. Conversely, they displayed higher levels of preference-adjusted social hostility toward out-group members (χ^2^ = 11.291, *df* = 1, *p* < 0.01). These findings suggest that even temporary group identities, formed in a sports setting, can lead to intergroup bias in adolescents’ social decision-making, promoting goodwill toward the in-group while restricting the autonomy of out-group members.

## 1. Introduction

Group identity refers to an individual’s sense of belonging to and identification with their group [1]. It influences prosocial behaviors that include helping [2], sharing [3,4], giving [5], charity [6,7], prosocial lying [8], resource allocation [2,9,10,11,12], and resolving social dilemmas [13,14]. Social mindfulness, a form of prosocial behavior [15,16,17,18,19,20,21], entails the attentive consideration of others’ needs and interests while respecting their autonomy preferences [22]. The skill to assess and the will to address situations of interdependence are needed to achieve social mindfulness [15,22,23,24,25]. Social mindfulness differs from other prosocial behaviors, such as resolving social dilemmas and economic games, primarily because of its minimal to negligible material costs and the challenge of ascertaining others’ preferences or desires [18,19,23,24,26,27,28]. Nevertheless, it is more aligned with prosocial behavior than altruism, given its lower cost and its focus away from collective welfare [29].

**Figure 1:**
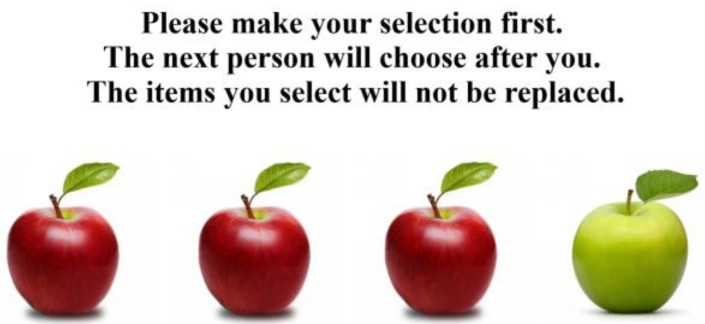
An example of the SoMi paradigm. Note: In the SoMi paradigm, one unique item (e.g., a green apple) and two or three non-unique items (e.g., red apples) are presented. Participants select an item, which is not replaced, and the next person chooses from the remaining items. Choosing a non-unique item is recorded as an instance of social mindfulness, whereas choosing a unique item is not. (Adapted from Van Lange and Van Doesum [23]; www.socialmindfulness.nl, accessed on 8 October 2022).

The SoMi paradigm has been applied extensively in research on social mindfulness [15,16,18,20,22,30]. Participants are presented with a series of decisions regarding three to four objects on a computer screen. In each trial, one object, designated as the unique object, differs slightly in an attribute (such as color, taste, or style) from the other objects, referred to as non-unique objects (Figure 1). Choosing a non-unique object is interpreted as yielding the choice to another individual and is recorded as an instance of social mindfulness. The computer program provides a reliable measure of social mindfulness levels. However, virtual options and real-life choices do not always align [31], potentially affecting the ecological validity of behavioral experiments [32,33]. Therefore, this study adapted the SoMi paradigm from a controlled laboratory setting to real-world scenarios [25].

The use of the SoMi paradigm has revealed that social mindfulness reflects an internal state and is also influenced by external factors. It aligns with prosocial value orientations [16,17,19,22,24]. This suggests that providing multiple choices for others could also represent a manifestation of social preference in resource allocation. Although its connection to agreeableness is debated [17,22,34], most studies correlate social mindfulness and prosocial personality traits, such as honesty–humility and altruism [16,17,22]. Conversely, social mindfulness and traits linked to antisocial behavior are negatively correlated [17]. Collectively, these studies propose that social mindfulness subtly signifies the decision-maker’s regard and modesty towards others.

Group identity creates contexts that differentiate co-decision-makers, making it a potential factor influencing the will to practice social mindfulness. In the hypothesized virtuous circle, cooperative relationships are linked with group formation. Group division and identification often lead in-group members to exhibit traits such as kindness, tolerance, and altruism, while out-group members tend to display indifference or hostility [35]. This suggests that group affiliation and antagonism can influence the willingness to cooperate, potentially affecting social mindfulness, particularly regarding the will aspect. Because perceived social mindfulness likely promotes cooperative behavior [36], it can reduce social distance among in-group members and increase exclusion between groups. Cooperative and prosocial behaviors within in-groups may be further strengthened, while opposition and conflict with out-groups could be exacerbated.

Long-term group identity influences social mindfulness decisions. Van Doesum et al. [37] found that foes received higher levels of social hostility compared to friends or strangers; opponents triggered social hostility, while teammates elicited social mindfulness. Unlike long-term organizations, temporary groups focus on completing specific tasks within a limited time frame [38]. These groups are common and crucial in real life, such as in managing short-term projects, undertaking temporary work, or forming ad hoc teams in response to natural disasters [39,40,41]. In the present study, temporary group identity refers to an identity formed without prior experiences with in-group or out-group members [42,43]. Temporary and permanent group relationships differ in social decision-making [44,45], although temporary groups may still quickly develop trust and shared goals [46]. To our knowledge, no research has yet explored the impact of group identity on social mindfulness within temporary groups.

The super-additive cooperation theory posits that the evolution of human social motives is shaped by mechanisms of both repeated interactions and group competition [13]. Team sports (e.g., basketball, soccer, and baseball) inherently involve both mechanisms: cooperation among in-group members and competition with out-group members. Even spectators of a basketball game displayed in-group favoritism, indicating that team sports activated group identity [47]. The present study also adopted the Minimal Group paradigm, a well-established method for examining group identity effects, to create temporary group affiliations. Participants were assigned to groups based on tasks involving inferences or similar preferences [42,43]. Previous research has demonstrated the applicability of this paradigm to adolescent populations [48], and its extension to a sports context is considered a viable approach for further exploration [49]. The present study measured social mindfulness levels toward in-group and out-group members within a team sports context.

Hypothetical and real prosocial behaviors are sometimes uncorrelated [31]. Additionally, peer presence can significantly enhance activity in the brain regions associated with social interaction [50]. Consequently, this study used the SoMi paradigm in real-life contexts to increase participants’ trust in the task and to better reflect actual decision-making, thereby enhancing ecological validity.

Adolescence is a critical period for the emergence of prosocial behaviors [9,51,52], during which social interactions play an important role in sharpening an individual’s ability to function in adulthood [53]. External factors, including parental and peer influences, significantly impact prosocial behavior in adolescents [54,55]. Studies suggest that cooperative or competitive contexts may influence adolescents’ decision-making, with cooperation potentially fostering positive behaviors and competition possibly reducing prosocial tendencies [56,57]. While self-reported prosocial behavior remains stable between the ages of 10 and 14 [58], the role of social mindfulness in adolescent decision-making remains unknown. Therefore, this study focused on adolescent participants to address this gap. The aim of the present study was to examine how group identity influences social mindfulness within the context of temporary groups. We hypothesized that adolescents divided into temporary groups would show greater social mindfulness toward in-group members and increased social hostility toward out-group members.

## 2. Materials and Methods

### 2.1. Participants

The sample size was calculated based on a pilot study that identified a large effect size, using G*Power 3.1.9.7 software with the following parameters: chi-square test, effect size = 0.5, 95% power, 5% significance level, and *df* = 1. A minimum sample size of 52 participants (26 per group) was indicated as necessary to achieve statistical significance among the groups [59,60]. The present study recruited 80 participants aged 10–14 years, who attended sessions with their parents and were informed of the research procedures. One participant did not complete the behavioral experiment due to food allergies, and five had missing questionnaire data. The final analysis included 74 participants: 60 males (81.08%) and 14 females (18.92%), with a mean age of 12.09 years (SD = 1.47). Additionally, post hoc analysis confirmed that this sample size was sufficient. All participants were free of major organic diseases, had good cognitive abilities, and had not previously participated in related experiments. This research was approved by the Ethical Committee of Yangzhou University (YXYLL-2024-020).

### 2.2. Design

This study employed a single-factor between-subjects design to examine social mindfulness decisions. The participants were randomly assigned to either the in-group decision (IG) or the out-group decision (OG) group.

### 2.3. Procedure

The day before the main experiment, the participants completed measurements of prosocial behavioral tendencies. On the experimental day, group identity was manipulated using a team sports context, referencing the grouping method from the Minimal Group paradigm. Ten participants selected wristbands in either blue or red; those with the same color were assigned to the same team. They then played a 10 min 5 vs. 5 basketball game, with the team scoring more points winning.

After the game, the referee announced the outcome and scores. The first assistant then took the participants to the laboratory for the social mindfulness behavioral task. The team members were randomly and alternately arranged—two blue, then two red—to distribute the participants evenly between the IG and OG and balance the win–loss distribution. IG participants received the instruction: “Please make your selection first. The next person will choose after you; the next person is your teammate. The items you select will not be replaced”. OG participants were told the following: “Please make your selection first. The next person will choose after you; the next person is your opponent. The items you select will not be replaced”. Following the task, the second assistant escorted the participants out of the laboratory to preclude communication between those who had and had not participated in the behavioral task.

### 2.4. Controlled Variables

Building on previous research, the present study included five controlled variables. The first was prosocial behavioral tendencies, which positively correlate with social mindfulness [16,17,19,22,24]. The second was age; although its relation to social mindfulness is debated [17,18,22,24,25,37,61], it is particularly important during adolescence, a period of rapid social cognitive development [62,63,64,65]. The third variable was the outcome (win, loss, or draw) of the team sports scenario, as it influences prosocial decision-making [66]. The fourth was participants’ preferences for items, given that unique or scarce items are highly desirable [67,68]; the alignment of participants’ decisions with their preferences was noted. Finally, the placement of the scarce items was considered, as it may potentially affect decision-making [69,70,71].

To ensure that differences in social mindfulness were not due to group variations, intergroup comparisons were conducted on factors such as age, scenario outcomes, item positioning, item preferences, and prosocial behavioral tendencies. The absence of statistically significant intergroup differences in these variables would demonstrate effective variable control and would indicate that differences in social mindfulness would be attributable to the experimental condition.

### 2.5. Measures

**Figure 2:**
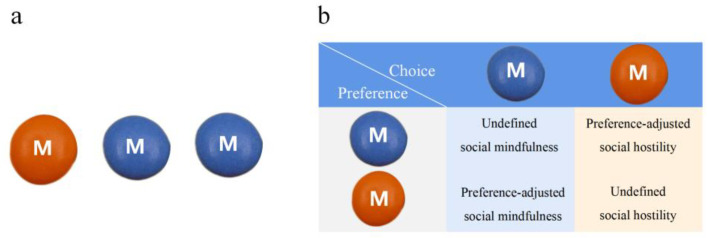
Illustration of adapted SoMi paradigm. Note: (**a**) Participants chose from three chocolate beans, one of which was of a different color. The in-group decision group participants had a teammate standing behind them, while the out-group decision group participants had an opponent standing behind them. (**b**) The decisions were categorized based on the participants’ choices and their reported preferences.

Based on the classic SoMi paradigm [22] and its real-life adaptation [25], this study used chocolate beans to match adolescent preferences (Figure 2a). In the laboratory, three beans were displayed, with one in a distinct color. Participants in the IG and OG followed specific instructions and made a single-trial decision. After choosing, they reported their true color preference (e.g., blue or orange). Selecting a non-unique item (e.g., a blue bean) was recorded as social mindfulness (Figure 2b). A choice that contradicted their true preference (e.g., choosing a blue bean when their true preference was orange) was categorized as preference-adjusted social mindfulness [16]. Conversely, selecting a unique item contrary to their preference was categorized as preference-adjusted social hostility (e.g., choosing an orange bean when their true preference was blue). The colors (orange, blue, yellow, green) and positions (left, center, right) were balanced and alternated across trials.

The Prosocial Tendencies Measure (PTM) by Carlo and Randall [72] was used to assess prosocial behaviors by applying six subscales: altruism, compliance, emotional, public, anonymous, and urgent. The participants rated items on a 5-point scale, with higher scores indicating stronger prosocial tendencies. Internal consistency reliabilities included altruism (0.708), compliance (0.778), emotional (0.755), public (0.738), anonymous (0.726), and urgent (0.561). Subscale correlations with the overall scale were altruism (0.494), compliance (0.758), emotional (0.801), public (0.800), anonymous (0.789), and urgent (0.803).

### 2.6. Statistical Analysis

Age and PTM scores were reported as means ± standard deviations, while other controlled variables (scenario outcomes, item positioning, and item preferences) and the three social mindfulness indicators were reported as frequencies. Independent sample *t*-tests compared age, PTM, and its subscale scores between the two groups, while chi-square analyses assessed differences in other controlled variables and the social mindfulness indicators.

## 3. Results

Intergroup comparisons of age, scenario outcomes, item positioning, item preferences, and prosocial behavioral tendencies revealed no significant differences, demonstrating effective variable control and indicating that observed differences in social mindfulness, preference-adjusted social mindfulness, and preference-adjusted social hostility were attributable to the experimental conditions.

### 3.1. Age

**Figure 3:**
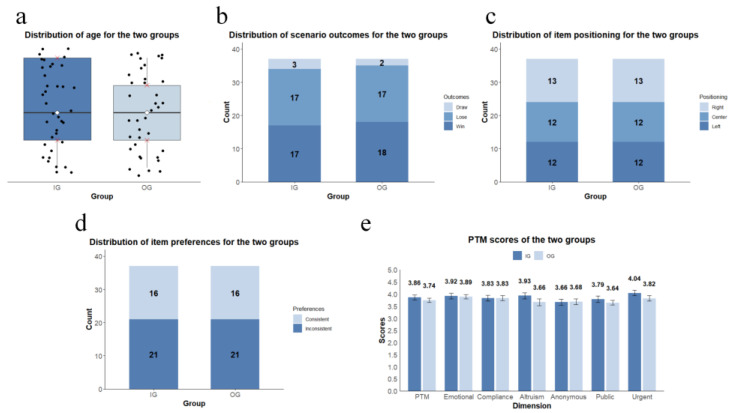
Distribution or scores of control variables for IG and OG. Note: (**a**) Distribution of age for the two groups. In the box plot, the white diamond represents the median, while the red crosses above and below the box indicate the first and third quartiles, respectively. (**b**) Distribution of scenario outcomes for the two groups. “Win”, “lose”, and “draw” represent the outcomes of the basketball game. (**c**) Distribution of item positioning for the two groups. “Left”, “center”, and “right” denote the positions of the uniquely colored chocolate bean among the three beans. (**d**) Distribution of item preferences for the two groups. “Consistent” indicates that participants’ choices matched their preferences, while “Inconsistent” indicates that participants’ choices differed from their preferences. (**e**) PTM scores of the two groups. Error bars represent the standard error. (IG: The in-group decision group; OG: the out-group decision group; PTM: the Prosocial Tendencies Measure).

The age distribution for both the in-group decision group (IG) and the out-group decision group (OG) is shown in Figure 3a. An independent sample *t*-test indicated no significant age difference between the IG (12.19 ± 1.49) and OG (12.00 ± 1.47) groups (*t* = 1.99, *df* = 72, *p* = 0.58).

### 3.2. Scenario Outcomes

Figure 3b provides an overview of the win, lose, and draw frequencies of the IG and OG groups in the basketball game. In the IG, 17 participants won, 3 drew, and 17 lost; in the OG, 18 participants won, 2 drew, and 17 lost. A chi-square analysis showed no significant intergroup differences (χ^2^ = 0.229, *df* = 2, *p* = 0.892).

### 3.3. Item Positioning

The frequency distribution of the unique item’s positions (left, center, right) during the social mindfulness task is depicted in Figure 3c. In both groups, 12 participants had the unique item on the left, 12 in the center, and 13 on the right. Item positioning did not differ significantly between the two groups (χ^2^= 0.000, *df* = 2, *p* = 0.999).

### 3.4. Item Preferences

Figure 3d depicts how frequently the chosen items aligned with actual preferences during the social mindfulness task. In both groups, 16 participants chose items consistent with their preferences, while 21 chose items inconsistent with their preferences. Preference alignment was similar between the two groups (χ^2^ = 0.000, *df* = 1, *p* = 0.999).

### 3.5. Prosocial Behavior Tendencies

The Prosocial Tendencies Measure (PTM) total and subscale scores, ranging from 0 to 5, are shown in Figure 3e. The IG group scored 3.86 ± 0.62, and the OG group scored 3.74 ± 0.52. The subscale scores were as follows: emotional (IG: 3.92 ± 0.71, OG: 3.89 ± 0.56), compliance (IG: 3.83 ± 0.69, OG: 3.83 ± 0.62), altruism (IG: 3.93 ± 0.75, OG: 3.66 ± 0.82), anonymous (IG: 3.66 ± 0.75, OG: 3.68 ± 0.70), public (IG: 3.79 ± 0.81, OG: 3.64 ± 0.59), and urgent (IG: 4.04 ± 0.67, OG: 3.82 ± 0.68). The independent sample *t*-tests revealed no significant intergroup differences in the total PTM score (*t* = 0.867, *df* = 72, *p* = 0.389) or any subscale scores: emotional (*t* = 0.218, *df* = 72, *p* = 0.828), compliance (*t* = 0.035, *df* = 72, *p* = 0.972), altruism (*t* = 1.472, *df* = 72, *p* = 0.145), anonymous (*t* = −0.096, *df* = 72, *p* = 0.924), public (*t* = 0.904, *df* = 72, *p* = 0.369), and urgent (*t* = 1.370, *df* = 72, *p* = 0.175).

### 3.6. Social Mindfulness

Intergroup comparisons of age, scenario outcomes, item positioning, item preferences, and prosocial behavioral tendencies revealed no significant differences, demonstrating effective variable control and indicating that observed differences in social mindfulness were attributable to the experimental conditions.

**Figure 4:**
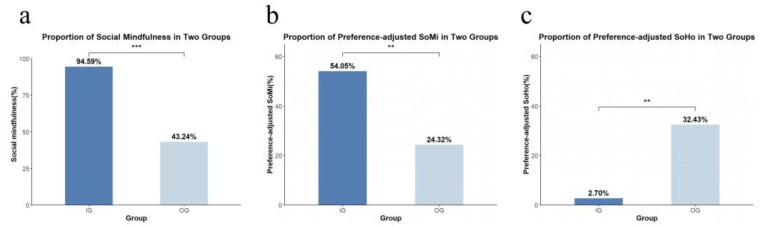
Proportion of SoMi, preference-adjusted SoMi and preference-adjusted SoHo for IG and OG. Note: (**a**) Proportion of social mindfulness in two groups. (**b**) Proportion of preference-adjusted SoMi in two groups. (**c**) Proportion of preference-adjusted SoHo in two groups. (IG: The in-group decision group; OG: the out-group decision group; SoMi: social mindfulness; SoHo: social hostility. ** *p* < 0.01, *** *p* < 0.001.)

A total of 35 IG group members exhibited social mindfulness, while two displayed non-social mindfulness, resulting in a social mindfulness rate of 94.59% (Figure 4a). In contrast, 16 OG group members exhibited social mindfulness, and 21 showed non-social mindfulness, yielding a social mindfulness rate of 43.24%. The rate of social mindfulness was significantly higher in the IG compared to the OG group (χ^2^= 22.774, *df* = 1, *p* < 0.001).

### 3.7. Preference-Adjusted Social Mindfulness

Twenty (54.05%) participants in the IG group exhibited preference-adjusted social mindfulness, compared to nine (24.32%) participants in the OG group (Figure 4b). The IG group showed a significantly higher rate of preference-adjusted social mindfulness (χ^2^ = 6.861, *df* = 1, *p* < 0.01).

### 3.8. Preference-Adjusted Social Hostility

Only one IG group member exhibited preference-adjusted social hostility (2.70%), compared to twelve participants in the OG group (32.43%) (Figure 4c), resulting in a significantly higher rate in the OG group (χ^2^ = 11.291, *df* = 1, *p* < 0.01).

## 4. Discussion

Under temporary group identity, adolescents exhibited intergroup bias in social mindfulness and hostility. Our study participants demonstrated higher social mindfulness toward in-group compared to out-group members, even when their choices contradicted their actual preferences. Conversely, they exhibited more pronounced preference-adjusted social hostility toward out-group members. These results were achieved after effectively controlling variables such as age, scenario outcomes, item positioning, item preferences, and prosocial behavioral tendencies. Following the reviewer’s suggestion, we conducted exploratory analyses using logistic regression to investigate the effects of group identity, PTM, and their interaction on social mindfulness. The results also revealed that only the main effect of group identity was significant.

Our findings may be understood through two components of social mindfulness, skill (perspective taking) and will (empathic concern), which together signal prosocial intentions [22,73]. While both are elements of empathy, they serve different functions. Adolescents have the requisite foundational skills to understand and make decisions regarding social mindfulness. With random group assignment and controlled variables, the skill levels between the two groups were comparable. We hypothesize that differences in will were the key factor behind the observed results.

Perspective taking is the ability to anticipate others’ behaviors and reactions, representing a key aspect of social functioning [74]. The theory of mind (ToM) is a related concept, involving the ability to attribute mental states—such as intentions, desires, and beliefs—to oneself and others [75]. ToM facilitates both the recognition that others have their own thoughts and feelings and the active assessment of their perspectives [76]. Even young children can recognize social mindfulness behaviors as thoughtful and prosocial actions [77,78]. The adolescents in both groups were within the age range during which prosocial behavior typically stabilizes (10–14 years), and did not display significant intergroup differences [58]. This homogeneity indicates that all participants had the foundational abilities for social mindfulness, with comparable levels of perspective taking and ToM.

Importantly, although adolescents have necessary decision-making skills, temporary group identities (in- or out-group) activated dissimilar levels of social mindfulness and hostility, reflecting varying levels of will [22]. After controlling for variables such as age and prosocial behavioral tendencies, the adolescents showed nearly a 95% probability of social mindfulness toward in-group members compared to less than 50% probability toward out-group members. This suggests that despite similar situational awareness and prosocial behavioral tendencies, differences in social mindfulness are likely due to variations in will, particularly social–emotional factors such as empathic concern. Even when accounting for preferences, the probability of preference-adjusted social mindfulness toward in-group members exceeded 50%, compared to under 25% toward out-group members. This indicates a greater willingness to prioritize social mindfulness for in-group members, with more than double the likelihood of forgoing personal preferences to grant choice to in-group compared to out-group members [16,34].

In contrast to the increased kindness exhibited toward in-group members, heightened hostility toward out-group members was identified through preference-adjusted social hostility. This form of hostility, which involves depriving others of choice even at the expense of one’s own preferences [23], manifested a 32.43% likelihood toward out-group members compared to only 2.70% toward in-group members. This result shows that temporary group identity influences social mindfulness by fostering both in-group favoritism and out-group derogation, as preference-adjusted social hostility provokes punitive behavior [79]. Our findings also support the concept that 10–14-year-old adolescents possess the cognitive abilities needed for social understanding and decision-making, such as perspective taking and ToM. The pronounced hostility toward out-group members indicates that adolescents can recognize and counteract others’ desires effectively.

The results of the present study align with previous research on prosocial behaviors within the Minimal Group paradigm [12,13,80], but this study offers new insights by extending the applicability of these findings to the domain of social mindfulness. Specifically, it demonstrates how temporary group identities, formed in a sports context, influence adolescents’ social decision-making, which has not been thoroughly explored in previous studies. Van Doesum, Van Prooijen, Verburgh, and Van Lange [37] found that among a sample of adolescent soccer players, opponents triggered social hostility, while teammates elicited social mindfulness. Both studies similarly found that individuals are more likely to favor in-group members by granting them choices, while limiting the options of out-group members.

The present study differed from previous research [37] in three major aspects, despite obtaining similar results. First and foremost was the manipulation of group identity. Previous studies explored long-term group identities, such as viewing in-group members as close friends and out-group members as long-standing rivals (Study 1), or categorizing in-group members as teammates and out-group members as top competitors (Study 2). In contrast, the present study investigated the effects of short-term, initial group identity, with participants assigned to temporary groups using a team sports scenario. Despite these differences, the similarity of observed patterns further supports the presence of intergroup bias in social mindfulness across various forms of group identity formation. Second, the present study adapted the SoMi paradigm to real-world settings, using actual items and wristbands for group identity, unlike previous research that used computer programs and club-specific items. This approach allowed participants to observe others’ identities from a distance, enhancing ecological validity [25,31,50]. Additionally, this study assessed both social mindfulness and social hostility, as well as preference-adjusted versions of these measures [16]. By controlling for preference effects, these indicators offer a clearer view of participants’ underlying intentions. Overall, despite variations in defining group identity, paradigms, and indicators, the consistent results demonstrate that the impact of group identity on social mindfulness is both broad and stable.

Adolescents showed higher levels of social mindfulness and lower levels of social hostility toward in-group members, suggesting greater attention to their needs and a higher willingness to provide them with choices [22]. This indicates a stronger prosocial tendency toward in-group members, who are more likely to benefit from actions that promote their welfare [29]. In contrast, lower levels of social mindfulness and increased social hostility toward out-group members suggest less regard for their needs, leading to behaviors that restrict their options and reduce prosocial tendencies. Despite adolescents’ ability to recognize interdependent situations, intergroup bias remained evident. This bias was particularly pronounced in the ‘will’ dimension of social mindfulness, indicating differing levels of willingness to respect the choices of in-group versus out-group members. Adolescents showed a greater readiness to act prosocially toward in-group members, while being less inclined to do so for out-group members, which aligns with the principles of social identity theory.

Social identity theory posits that individuals recognize their membership in specific social groups and derive emotional and value-based significance from their group identity [81]. In social mindfulness, in-group favoritism enhances the status of the in-group, thereby satisfying individuals’ need for positive self-esteem by benefiting in-group members [1,35]. Therefore, individuals maintain higher social mindfulness toward in-group members, even at the expense of their own preferences. While individuals can make social mindfulness decisions, their willingness to act toward in- and out-group members varies significantly. Participants, sensing a strong affiliation with the in-group, seek to elevate their own group, while demeaning out-group members.

The findings suggest that adolescents display distinct prosocial tendencies based on group identities in temporary group settings. Such identity distinctions are prevalent in real-life situations and should be addressed in group activities. First, educators could encourage cooperation among in-group members while reducing opposition or hostility toward out-group members. Second, structuring group arrangements to promote intergroup mobility may help prevent prolonged adversarial relationships. These approaches could promote healthier peer relationships and cultivate more positive attitudes toward future competitive interactions.

This study had several limitations. This study employed team sports—a context that combines cooperation and competition—to activate group identity. Notably, experiences of cooperation and competition may play distinct or overlapping roles in in-group favoring and out-group derogation. This research did not empirically separate the effects and interactions of these two elements. Future studies should explore their independent influences on social mindfulness. Additionally, sex differences also influence social decision-making, particularly in specific categories of prosocial behavior [82]. The role of sex differences in social mindfulness is debated: one study found that females exhibit higher levels of social mindfulness compared to males [22], while others report no significant influence of sex on social mindfulness decision-making [17,19]. We conducted chi-square analyses by sex within both groups and found no significant differences. However, the high level of sex homogeneity in the sample presents a potential limitation.

## 5. Conclusions

This study found that temporary group identities impact adolescents’ social mindfulness decisions, resulting in intergroup bias. Adolescents displayed higher levels of both social mindfulness and preference-adjusted social mindfulness toward in-group members (in-group favoritism) while showing increased preference-adjusted social hostility toward out-group members (out-group derogation). These findings highlight the importance of understanding how temporary group dynamics influence adolescents’ social behaviors and the implications for promoting positive intergroup relations.

## Data Availability

The original contributions presented in this study are included in this article, and further inquiries can be directed to the corresponding authors.
